# Supplementary material for: Content-rich biological network constructed by mining PubMed abstracts
Source: BMC Bioinformatics. 2004 Oct 8;5:147. doi: 10.1186/1471-2105-5-147 (PMC528731; doi:10.1186/1471-2105-5-147)
Supplement: Additional File 2 — The original results of the above study (non-essential files are deleted to keep the file size under the limit set by BMC bioinformatics). [file 1471-2105-5-147-S2.bz2 › chilibotAdditionalFile2/dip05/368ID9045692E6042/html/FGFR3_FGF4.html]

 


 **FGFR3** and **FGF4** 
  
Found 17 abstracts in PubMed, retrieved 05.  
 

 What does Google say? 
 PDF only 
| .edu only 

---

- Mol Cell Neurosci, 2003   **Possible involvement of a fibroblast growth factor 9 FGF9 FGF receptor 3 mediated pathway in adult pig retinal ganglion cell survival in vitro.** .
  The expression and potential roles of fibroblast growth factors FGF and their cognate FGF receptors FGFR in adult mammalian retinal ganglion cells RGC are poorly known.
  We show that FGFR 3 **[FGFR3]** and FGFR 4 are especially pronounced on RGC and amacrine cell bodies in adult pig inner retinae both in vivo and in vitro.
  Western blotting revealed distinct profiles for each receptor.
  Expression of each FGFR and effects of the preferred ligand for FGFR 3 **[FGFR3]**, FGF9, upon RGC survival and neurite outgrowth were examined in primary retinal cell cultures whereas there was no stimulation of neuritogenesis, RGC survival was promoted in a dose dependent manner ED 50 approximately 500 pg ml, mean maximal increase of 60% and could be completely blocked by addition of FGF9 neutralising antibody.
  Experiments with three additional FGF FGF1, FGF2, and **FGF4** showed no stimulation of RGC survival above control levels.
  Taken together, these data suggest that the ligand receptor couple FGF9 FGFR 3 **[FGFR3]** may function to promote survival of adult mammalian RGC, and their application might be beneficial in retinal degenerative diseases such as glaucoma.

  - Dev Biol, 2003   **Fibroblast growth factor FGF 4 **[FGF4]** can induce proliferation of cardiac cushion mesenchymal cells during early valve leaflet formation.** .
    While much has been learned about how endothelial cells transform to mesenchyme during cardiac cushion formation, there remain fundamental questions about the developmental fate of cushions.
    In the present work, we focus on the growth and development of cushion mesenchyme.
    We hypothesize that proliferative expansion and distal elongation of cushion mesenchyme mediated by growth factors are the basis of early valve leaflet formation.
    As a first step to test this hypothesis, we have localized fibroblast growth factor FGF 4 **[FGF4]** protein in cushion mesenchymal cells at the onset of prevalve leaflet formation in chick embryos Hamburger and Hamilton stage 20 25 .
    Ligand distribution was correlated with FGF receptor FGFR expression.
    In situ hybridization data indicated that **FGFR3** mRNA was confined to the endocardial rim of the atrioventricular AV cushion pads.
    . . . FGFR2 was expressed exclusively in cushion mesenchymal cells.
    FGFR1 expression was detected in both endocardium and cushion mesenchyme as well as in myocardium.
    To determine whether the FGF pathways play regulatory roles in cushion mesenchymal cell proliferation and elongation into prevalvular structure, FGF 4 **[FGF4]** protein was added to the cushion mesenchymal cells explanted from stage 24 25 chick embryos.
    A significant increase in proliferative ability was strongly suggested in FGF 4 **[FGF4]** treated mesenchymal cells as judged by the incorporation of 5 bromodeoxyuridine BrdU .
    To determine whether cushion cells responded similarly in vivo, a replication defective retrovirus encoding FGF 4 **[FGF4]** with the reporter, bacterial beta galactosidase was microinjected into stage 18 chick cardiac cushion mesenchyme along the inner curvature where AV and outflow cushions converge.
    As compared with vector controls, overexpression of FGF 4 **[FGF4]** clearly induced expansion of cushion mesenchyme toward the lumen.
    To further test the proliferative effect of FGF 4 **[FGF4]** in cardiac cushion expansion in vivo ovo , FGF 4 **[FGF4]** protein was microinjected into stage 18 chick inner curvature.
    An assay for BrdU incorporation indicated a significant increase in proliferative ability in FGF 4 **[FGF4]** microinjected cardiac cushion mesenchyme as compared with BSA microinjected controls.
    Together, these results suggest a role of FGF 4 **[FGF4]** for cardiac valve leaflet formation through proliferative expansion of cushion mesenchyme.

    - Exp Eye Res, 2003   **Human RPE cells express the FGFR2IIIc and FGFR3IIIc splice variants and FGF9 as a potential high affinity ligand.** .
      The expression of splice variants of FGF receptors, which differ in the third Ig domain, was investigated in retinal pigment epithelium RPE cells in vitro and in vivo.
      This region of the protein determines ligand binding specificity.
      Additionally, the expression of potential ligands for these receptors was investigated.
      Expression of FGF receptor transcript alternative splicing was analyzed by RT PCR Southern analysis in RPE cells in vitro and in vivo.
      The expression of FGFs by RT PCR, in situ hybridization, and immunohistochemistry in sections of the human posterior pole was also investigated.
      The ARPE 19 cell line expresses only the FGFR2IIIc splice variant and does not express any **FGFR3** splice variants in vitro.
      Two in vivo samples exhibited expression of the FGFR2IIIc and FGFR3IIIc splice variants and no evidence of the corresponding IIIb splice variant.
      The results from previous studies for these receptors imply that FGF9 or **FGF4** could act as ligands.
      We demonstrated that FGF9 is expressed in a subpopulation of the RPE, as well as photoreceptors and other neurons of the retina.
      **FGF4** was not detected by RT PCR analysis in RPE cells in vitro.
      These data suggest that FGF9 may be an autocrine paracrine factor in the outer retina.

      - Dev Dyn, 2002   Dach1 is a mouse homologue of the Drosophila dachshund gene, which is a key regulator of cell fate determination during eye, leg, and brain development in the fly.
        We have investigated the expression and growth factor regulation of Dach1 during pre and postnatal skeletal development in the mouse limb to understand better the function of Dach1.
        Dach1 was expressed in the distal mesenchyme of the early embryonic mouse limb bud and subsequently became restricted to the tips of digital cartilages.
        Dach1 protein was localized to postmitotic, prehypertrophic, and early hypertrophic chondrocytes during the initiation of ossification centers, but Dach1 was not expressed in growth plates that exhibited extensive ossification.
        Dach1 colocalized with Runx2 Cbfa1 in chondrocytes but not in the forming bone collar or primary spongiosa.
        Dach1 also colocalized with cyclin dependent kinase inhibitors p27 and p57 in chondrocytes of the growth plate and in the epiphysis before the formation of the secondary ossification center.
        Because fibroblast growth factors , bone morphogenetic proteins , and hedgehog molecules regulate skeletal patterning of the limb bud and chondrocyte maturation in developing endochondral bones, we investigated the regulation of Dach1 by these growth and differentiation factors.
        Expression of Dach1 in 11 days postcoitus mouse limb buds in organ culture was up regulated by implanting beads soaked in FGF , 8, or 9 but not FGF10.
        BMP4 soaked beads down regulated Dach1 expression.
        . . . Shh and bovine serum albumin had no effect.
        Furthermore, **FGF4** or 8 could substitute for the apical ectodermal ridge in maintaining Dach1 expression in the limb buds.
        Immunolocalization of FGFR2 and **FGFR3** revealed overlap with Dach1 expression during skeletal patterning and chondrocyte maturation.
        We conclude that Dach1 is a target gene of FGF signaling during limb skeletal development, and Dach1 may function as an intermediary in the FGF signaling pathway regulating cell proliferation or differentiation.

        - Cleft Palate Craniofac J, 2002   **Toward pathogenesis of Apert cleft palate FGF, FGFR, and TGF beta genes are differentially expressed in sequential stages of human palatal shelf fusion.** .
          OBJECTIVE Critical cellular events at the palatal medial edge epithelium MEE occur in unperturbed mammalian palatogenesis, the molecular control of which involves a number of growth factors including transforming growth factor beta 3 TGF beta 3 .
          Apert syndrome is a monogenic human disorder in which cleft palate has been significantly correlated to the fibroblast growth factor receptor FGFR 2 Ser252Trp mutation.
          We report the relative expression of these genes in human palatogenesis.
          METHODS The expression of the IgIIIa b and IgIIIa c transcript isoforms of FGFR2 and the proteins FGFR1, FGFR2, and **FGFR3** was studied in situ throughout the temporospatial sequence of human palatal shelf fusion and correlated with the expression of TGF beta 3.
          In addition, the immunolocalization of the ligand FGFs , and 7 was undertaken together with the intracellular transcription factor STAT1, which is activated by FGFR signaling.
          RESULTS FGFRs are differentially expressed in the mesenchyme and epithelia of fusing palatal shelves, in domains overlapping those of their ligands **FGF4** and FGF2 but not FGF7.
          Coexpression is seen with TGF beta 3, which is implicated in MEE dynamics and FGF and FGFR upregulation, and STAT1, an intracellular transcription factor that mediates apoptosis.
          CONCLUSIONS The coregulation of molecules of the FGFR signaling pathway with TGF beta 3 throughout the stages of human palatal fusion suggests their controlling influence on apoptosis and epitheliomesenchymal transdifferentiation at the MEE.
          Experimental evidence links FGFR2 IgIIIa b loss of function with palatal clefting, and these correlated data suggest a unique pathological mechanism for Apert cleft palate.
